# Adherence to the Norwegian dietary recommendations in a multi‐ethnic pregnant population prior to being diagnosed with gestational diabetes mellitus

**DOI:** 10.1002/fsn3.1248

**Published:** 2019-10-22

**Authors:** Lisa Garnweidner‐Holme, Liv Elin Torheim, Lena Henriksen, Iren Borgen, Sigrid Holmelid, Mirjam Lukasse

**Affiliations:** ^1^ Faculty of Health Sciences Institute of Nursing and Health Promotion OsloMet – Oslo Metropolitan University Oslo Norway

**Keywords:** ethnicity, Food Frequency Questionnaire, gestational diabetes mellitus, maternal diet

## Abstract

Maternal diet is a modifiable risk factor for the development of gestational diabetes mellitus (GDM). Even though pregnant women are considered to be motivated to eat healthy, previous research found unhealthy eating patterns among some ethnic and lower socio‐economic status groups. This cross‐sectional study assessed adherence to national dietary recommendations prior to GDM diagnosis in a multi‐ethnic population comprising 237 pregnant women. Participants were diagnosed with GDM after performing a two‐hour oral glucose tolerance test ≥ 9 mmol/L. Participants answered a 41‐item Food Frequency Questionnaire about dietary habits prior to being diagnosed with GDM from October 2015 to March 2018. Their scores were based on adherence to the recommended intake in each food group and summed into a Healthy Diet Score (HDS). Results showed low adherence to national dietary recommendations. A significantly higher proportion of non‐native Norwegian‐speaking women had a high HDS compared with native Norwegian‐speaking women. Participants with a normal prepregnancy weight were more likely to have a high HDS compared with overweight or obese participants. Participants showed low adherence to the recommendations for whole grains, vegetables, and fruits and berries, and a relatively low proportion adhered to the recommendations for intakes of fish, red/processed meat, and ready‐made meals. However, the food group intakes varied by country of birth. Given the increase in women with GDM and the emerging evidence that maternal diet is a modifiable risk factor for GDM, effective nutrition communication strategies in antenatal care are urgently needed.

## Graphical abstract

This cross‐sectional study assessed adherence to national dietary recommendations prior to GDM diagnosis in a multi‐ethnic population comprising 237 pregnant women. Results showed low adherence to national dietary recommendations. A significantly higher proportion of non‐native Norwegian‐speaking women had a high Healthy Diet Score compared with native Norwegian‐speaking women.

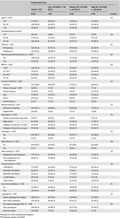

## 1 INTRODUCTION

Maternal diet is a modifiable risk factor for gestational diabetes mellitus (GDM) (Kim, 2014; Shin, Lee, & Song, 2015). GDM is defined as glucose intolerance with first onset or recognition in pregnancy (World Health Organization, 2013). The development of GDM not only increases the risk of complications during pregnancy and birth (Neiger, 2017) but is associated with increased long‐term risk of type 2 diabetes mellitus (T2DM) and cardiovascular disease for the mother and child (Shah, Retnakaran, & Booth, 2008). For instance, women who have had GDM are, at least, at a seven‐fold increased risk of developing T2DM compared to those with euglycemic pregnancies (Bellamy, Casas, Hingorani, & Williams, 2009). Furthermore, children born to mothers with GDM already face a higher risk of developing T2DM at a younger age and obesity and cardiovascular disease later in life (Gluckman, Hanson, & Buklijas, 2010). Thus, efforts to prevent GDM are paramount.

The prevalence of GDM is increasing worldwide and ranges from 1% to 20% globally depending on the screening procedure and population characteristics (Rani & Begum, 2016). The prevalence is increasing in Norway as well and was estimated at 5.2% in 2016 (Medical Birth Registry Norway, 2018). However, a cohort study in Groruddalen, a district in Oslo, identified GDM in 13% of all women, 11% in ethnic Norwegians, and 12%–17% in groups of non‐European origin (Jenum et al., 2012). Women from South Asian and African backgrounds tend to develop GDM at a lower BMI and age compared with white Europeans (Makgoba, Savvidou, & Steer, 2012). Other risk factors for developing GDM include overweight and obesity, advanced maternal age, a family history of diabetes, and GDM in a previous pregnancy (Schneider et al., 2011). As a large population‐based cohort study in the Netherlands found, when adjusting for ethnicity, family history of diabetes, and parity, low‐educated women faced an increased risk of GDM mainly due to higher rates of overweight and obesity (Bouthoorn et al., 2015).

Dietary intervention studies on the prevention of GDM are abundant but there is, as yet, no conclusive evidence on what constitutes the optimal dietary pattern for the prevention of GDM (Olmedo‐Requena et al., 2019; Schoenaker, Mishra, Callaway, & Soedamah‐Muthu, 2016; Shepherd et al., 2017). In the Norwegian guidelines for antenatal care, health professionals are encouraged to provide women with information about a healthy, varied diet in line with the national dietary recommendations in order to prevent foodborne diseases (Directorate for Health & Social Affairs, 2005). In particular, women are encouraged to eat whole‐grain products, fruits and vegetables, lean dairy, and meat products and to limit their sugar and salt intakes. However, studies indicate that pregnant women received little nutrition‐related advice in antenatal care, and participants from immigrant backgrounds appeared to be confused about dietary advice that was incongruent with their original food culture (Garnweidner, Sverre Pettersen, & Mosdol, 2013; Szwajcer, Hiddink, Koelen, & van Woerkum, 2005).

Although pregnant women are considered to be motivated to eat healthy, several studies in different countries found unhealthy eating patterns among pregnant women (Shapiro et al., 2016; Zhu et al., 2018; von Ruesten et al., 2014). According to a contemporary multiracial prospective cohort study, the majority (79%) of pregnant women did not adhere to the Dietary Guidelines for Americans (Zhu et al., 2018). Moreover, ethnic differences in maternal dietary patterns were found (Sommer et al., 2013). A cross‐sectional study among 757 pregnant women in Norway found that all non‐European women, as compared to Europeans, were more likely to have the unhealthier dietary pattern (Sommer et al., 2013). Given the increased risk of GDM in women of African and South Asian origin, it is important to gain more knowledge about possible ethnicity‐related differences in the dietary quality of pregnant women.

Thus, the aim of this study was to assess adherence to national dietary recommendations in a multi‐ethnic pregnant population prior to being diagnosed with GDM.

## 2 METHODS

### 2.1 Design and study sample

A cross‐sectional study was performed using baseline data from the Pregnant+ study, a randomized controlled trial (RCT) among pregnant women with GDM (Borgen et al., 2017) (ClinicalTrials.gov/NCT02588729). Data were collected from October 2015 to April 2017 at five diabetic outpatient clinics (DOCs) in the Oslo region in Norway. Participants were recruited consecutive as they came to the DOC. Health professionals at the DOCs identified pregnant women with GDM and checked their eligibility for participating in the study. Of 774 participants assessed for eligibility, 238 participated and the data from 237 could be used for this study.

Data were collected from 237 pregnant women, all of whom were diagnosed with GDM after performing a two‐hour oral glucose tolerance test (OGTT) ≥ 9 mmol/L. The period between when participants received the diagnosis and filled out the questionnaire varied from one to seven days. The definition of GDM was in accordance with the national guidelines for antenatal care and the WHO (Legeforeningen, 2008; World Health Organization, 2013). To be included in the study, women had to have a smartphone, be 18 years or older, and be at a gestational age of at least 33 weeks. The women had to be capable of filling out the questionnaire in either Norwegian, Somali, or Urdu. Only 14 women filled out the questionnaire in either Urdu or Somali. Participants were excluded from the study if they had a twin pregnancy. In addition, women with celiac disease or lactose intolerance were excluded because they must follow special diets (Borgen et al., 2017). The study was approved by the Norwegian Social Science Data Services (ID number 2014/38942), and the patient privacy protections boards governing each of the recruiting sites. Written consent was obtained from all participants.

### 2.2 Measures

Women answered the questionnaires on an electronic tablet at their first consultation at a DOC. Participants were asked to complete a 41‐item Food Frequency Questionnaire (FFQ) reporting their dietary habits prior to being diagnosed with GDM. The FFQ included the following food groups: beverages, milk and dairy products, bread and grain, fruit and vegetables, snacks, meat, and processed foods. Answers to the questions on frequency of intake ranged from 0 (never) to 9 (several times daily). The FFQ was based on the Fit for Delivery study and was shown to have an adequate level of test–retest reliability (Øverby, Hillesund, Sagedal, Vistad, & Bere, 2015). The FFQ in Somali and Urdu was pilot tested among Somali and Pakistani Norwegian women. Participants were given scores based on adherence to the recommended frequency of intake, which varied among the different food groups. The adequacy of intake of healthy foods and moderation of intake of less healthy foods were taken into consideration (Brosig, Burggraf, Teuber, & Meier, 2018), and points of 0, 5, or 10 were given for the level of adherence to each food group (Table 2). The scores were summed into a Healthy Diet Score (HDS), which was categorized into tertiles of “low” (<40 HDS), “medium” (45–60 HDS), and “high” (65–120 HDS) adherence to the recommendations.

### 2.3 Statistical analysis

Statistical analyses were performed with SPSS for IBM statistical software package version 24.0 (IBM Corporation, Armonk, NY, USA). Cross‐tabulations with Pearson's chi‐square tests were used to calculate percentages and assess differences in background characteristics and adherence to a healthy diet by HDS tertiles. Multinomial logistic regression analysis was used to examine the relationship between the different HDS (high, medium, and low) and background characteristics. Univariable models were performed first, with the HDS as the dependent variable. All variables in the preliminary univariable models were included in a multivariable model if they were significantly associated with one or more of the different levels in the HDS. Both language (categorized as non‐native and native Norwegian speaking) and country of birth (categorized as Norway, Western Europe and United States; Eastern Europe; Asia; Africa; and South America) were significantly associated with the HDS. Country of birth was retained in the final model to show more nuanced differences. A *p*‐value below .05 was the level of inclusion.

Differences in frequency of food group intakes by categories of BMI and by country of birth were analyzed via an analysis of variance (ANOVA). Differences in intakes between two countries/regions were analyzed via a Student *t* test.

## 3 RESULTS

A total of 237 women were included in the study. The majority were between 24 and 32 years old (76.8%). Mean gestational age when filling out the questionnaire was 26.7 weeks (*SD* = 4.9). Mean prepregnancy BMI was 26.7 (*SD* = 5.7) (not in tables). Table 1 contains background characteristics for the whole sample and according to the tertiles of HDS. A total of 108 (45.6%) participants were native Norwegian speakers, and 129 (54.4%) were non‐native Norwegian speakers. Nearly 25% (24.1%) were born in Eastern Europe and Asian countries. A total of 12.2% were born in African countries, while 5.9% were born in Western countries, including the United States, and 2.1% in South American countries. The proportion of women with poor language skills was very small (7.4%).

**Table 1:** Background characteristics in the total sample and by Healthy Diet Score, *N* = 237

	Healthy Diet Score				
	Total *N* = 237	Low (<40 HDS) *n* = 68 (28.7)	Medium (45–60 HDS) *n* = 87 (36.7)	High (65–120 HDS) *n* = 82 (34.6)	*p*‐value
	*N* (%)	*n* (%)	*n* (%)	*n* (%)	
Age (*n* = 237)					.42
<29	57 (24.1)	18 (26.5)	23 (26.4)	16 (19.5)	
30–37	128 (54.0)	32 (47.1)	45 (51.7)	51 (62.2)	
>38	52 (21.9)	18 (26.5)	19 (21.8)	15 (18.3)	
Gestational age in weeks					
<17	18 (7.6)	6 (8.8)	8 (9.2)	4 (4.9)	.36
18–24	37 (15.6)	11 (16.5)	17 (19.5)	9 (11.0)	
24–32	182 (76.8)	51 (75.0)	62 (71.3)	69 (84.1)	
Parity (*n* = 237)					.61
Primiparous	110 (46.4)	35 (51.5)	39 (44.8)	36 (43.9)	
Multiparous	127 (53.6)	33 (48.5)	48 (55.2)	46 (56.1)	
Previous gestational diabetes (*n* = 162^b^)					.87
No	118 (72.8)	33 (73.3)	47 (74.6)	38 (70.4)	
Yes	44 (27.2)	12 (26.7)	16 (25.4)	16 (29.6)	
BMI (*n* = 234)					.02
<24.9	101 (43.2)	21 (31.3)	35 (40.7)	45 (55.6)	
25–29.9	74 (31.5)	27 (40.3)	25 (29.1)	22 (26.8)	
30–34.9	37 (15.6)	9 (13.4)	16 (18.6)	12 (14.6)	
35–45	22 (9.4)	10 (14.9)	10 (11.6)	2 (2.5)	
Country of birth (*n* = 237)					.14
Norway	111 (46.8)	38 (55.9)	45 (51.7)	28 (34.1)	
Western Europe + USA	14 (5.9)	5 (7.4)	3 (3.4)	7 (7.3)	
Eastern Europe	21 (24,1)	4 (5.9)	6 (6.9)	11 (13.4)	
Asia	57 (24.1)	15 (22.1)	23 (26.4)	19 (23.2)	
Africa	29 (12,2)	5 (7.5)	8 (9.2)	16 (19.5)	
South America	5 (2.1)	1 (1.5)	2 (2.3)	2 (2.4)	
Marital status (*n* = 237)					.73
Married/co‐habiting	221 (93.2)	64 (94.1)	82 (94.3)	75 (91.5)	
Single/Other	16 (6.8)	4 (5.9)	5 (5.7)	7 (6.8)	
Education (*n* = 237)					.92
Primary school/Non education	23 (9,7)	8 (11,8)	6 (6.9)	9 (11.0)	
High school	56 (23.6)	18 26.5)	21 (24.1)	17 (20.7)	
College or University <4 years	59 (24.9)	16 (23,5)	22 (25.3)	21 (25.6)	
College or University ≥4 years	99 (41.8)	26 (38.2)	38 (43.7)	35 (42.7)	
Smoking (*n* = 237)					.45
No	235 (98.7)	68 (100)	85 (97.7)	81 (98.8)	
Yes	3 (1.3)	0 (0.0)	2 (2.3)	1 (1.2)	
Wet tobacco (*n* = 237)					.02
No	234 (98.7)	65 (95.6)	87 (100)	82 (100)	
Yes	3 (1.3	3 (4.4)	0 (0.0)	0 (0.0)	
Main activity (*n* = 237)					.29
Employed or self‐employed	179 (75.5)	54 (79.4)	68 (78.2)	57 (69.5)	
Not employed or not self‐employed	58 (24.5)	14 (20.6)	19 (21.8)	25 (30.5)	
Joined income (*n* = 237)					.32
≤599.000 kr.	77 (32.9)	22 (32.4)	27 (31.0)	29 (35.4)	
600.000–799.000 kr.	30 (12.7)	10 (14.7)	9 (10.3)	11 (13.4)	
800.000–999.000 kr.	50 (21.1)	18 (26.5)	17 (19.5)	15 (11.0)	
≥1.000.000 kr.	39 (16.5)	9 (13.2)	21 (24.1)	9 (11.0)	
I don't know	40 (16.9)	9 (13.2)	13 (14.9)	12 (22.0)	
Economic hardship (*n* = 231)					.91
No	72 (30.4)	19 (30.2)	26 (30.2)	27 (32.9)	
Yes	159 (67.1)	44 (69.8)	60 (67.1)	55 (67.1)	
Language (*n* = 237)					.01
Native speaking Norwegians	108 (45.6)	39 (57.4)	42 (48.3)	27 (32.9)	
Non‐native speaking	129 (54.4)	29 (42.6)	45 (51.7)	55 (67.1)	
Norwegian language skills^a^ (*n* = 128)					.01
Very good/good	109 (85.2)	23 (82.1)	35 (77.8)	51 (92.7)	
Poor/very poor	19 (14.8)	5 (17.9)	10 (22.2)	4 (7.3)	

a Among non‐native speaking Norwegians.

b Primiparous women excluded.

Participants were categorized as having low (28.7%), medium (36.7%), or high (34.6%) HDS. A significantly higher proportion of non‐native Norwegian‐speaking women had a high HDS compared with native Norwegian‐speaking women (Table 1). Furthermore, compared with overweight or obese women, a significantly higher proportion of participants with normal weight prepregnancy had high HDS. Dietary scores were not associated with women's socio‐economic status and education.

Concerning the intake of food groups included in the HDS, participants showed low adherence to the recommendations for whole grains, vegetables, and fruits and berries (Table 2). A relatively low proportion adhered to the recommendations for intakes of fish, red/processed meat, and ready‐made meals.

**Table 2:** Description of the Healthy Diet Score: Norwegian dietary recommendations, related Food Frequency Questionnaire questions, cutoff values, and distribution for each component, *N* = 237

Component (food group)	Dietary recommendations	Related FFQ‐question(s): How often did you…^a^	Cutoff values^b^	Score	Distribution *N* = 237	
					*n*	%
Whole grain	4 portions/day	Eat items of whole‐grain flour	<1 t/d	0	109	46
			1 t/d	5	105	43
		Eat whole‐grain pasta/rice	>1 t/d	10	23	10
Vegetables<>	≥2.5 portions/day	Eat vegetables for dinner	<1 t/d	0	124	52
		Eat vegetables for other meals	1 t/d	5	101	63
			>1 t/d	10	12	5
Fruits and berries	≥2.5 portions/day	Eat banana, litchi, mango, grapes	< 1 t/d	0	141	59
		Eat other fruits	1 t/d	5	87	37
			>1 t/d	10	9	4
Milk (low fat)	3 portions/day	Drink low fat or skimmed milk	0–3 t/w	0	111	47
			4–6 t/w	5	40	17
			≥1 t/d	10	86	36
Fish	2–3 times/week	Eat fish for dinner	<1 t/w	0	31	13
			1 t/w	5	64	27
			≥ 2 t/w	10	142	60
Beans and lentils	Estimated: 2 times/week	Eat beans or lentils	<1 t/w	0	129	54
			1 t/w	5	27	11
			≥2 t/w	10	81	34
Vegetable oils	PUFA 5–10 E%	Use vegetable oil when cooking	<3 t/w	0	18	8
	MUFA 10–20 E%		4–6 t/w	5	8	3
			≥7 t/w	10	211	89
Red/processed meat	Max 500 g/w	Eat red and/or processed meat	≥2 t/w	0	162	68
			1 t/w	5	39	17
			<1 t/w	10	36	15
Ready‐made meals	Max 6 g salt/day	Eat ready‐made meals for dinner («bagged food», pizza, French fries etc))	≥1 t/w	0	105	44
			1 t/w	5	87	37
			<1 t/w	10	45	19
Salt	Max 6 g salt/day	Use table salt on your food	>2 t/w	0	103	43
		Eat potato chips or other salty snacks	1–2 t/w	5	80	34
			<1 t/w	10	54	23
Sugar	Max 10 E%	Put sugar on your food	≥8 t/w	0	130	55
		Eat sweets/chocolate/candy	3–7 t/w	5	66	28
		Eat sweet biscuits, baked goods, ice cream, pudding	<3 t/w	10	41	17
		Eat yoghurt with added sugar				
		Drink sugar‐sweetened beverages				
Saturated fat	Max 10 E%	Use butter or ghee when cooking	≥2 t/w	0	161	68
			1 t/w	5	17	7
			<1 t/w	10	59	25

a FFQ asking retrospectively what the woman ate prior to being diagnosed with gestational diabetes.

b The frequency of intake in times (*t*) per day (d) or week (w).

When adjusting for country of birth, obese women (BMI 35–45) had a nine‐fold increase in the odds ratio of having a low HDS compared with normal weight women (AOR = 9.22, 95%; CI 1.89–47.17) (Table 3). Women born in an African country were less likely to have a low or medium HDS compared with Norwegian‐born women when adjusting for BMI (AOR = 0.24 [0.07–0.74] and 0.32 [0.12–0.88]).

**Table 3:** Odds ratios (and 95% confidence intervals) for adherence to a healthy diet by body mass index and country of birth, *N* = 237

	Healthy diet score			
	Medium score versus high		Low score versus high	
	*n* = 87 (36.7)		*n* = 68 (28.7)	
	OR	AOR	OR	AOR
*BMI*				
18.5–24.9	ref	ref	ref	ref
25–29.9	1.46 (0.70–3.01)	1.44 (0.68–3.00)	2.63 (1.22–5.65)	2.68 (1.22–5.88)
30–34.9	1.71 (0.71–4.09)	1.64 (0.67–4.05)	1.61 (0.59–4.40)	1.44 (0.51–4.09)
35–45	6.43 (1.32–31.24)	5.97 (1.19–29.86)	10.71 (2.15–53.28)	9.22 (1.80–47.17)
*Country of birth*				
Norway	ref	ref	ref	ref
Western Europe + USA	0.31 (0.72–1.35)	0.34 (0.07–1.43)	0.61 (0.17–2.22)	0.69 (0.18–2.67)
Eastern Europe	0.34 (0.11–1.02)	0.43 (0.12–1.32)	0.27 (0.07−0.0.93)	0.34 (0.09–1.22)
Asia	0.75 (0.35–1.63)	0.90 (0.14–1.32)	0.58 (0.25–1.34)	0.68 (0.28–1.61)
Africa	0.31 (0.12–0.82)	0.32 (0.12–0.88)	0.23 (0.08–0.70)	0.24 (0.07–0.74)
South America	0.62 (0.83–4.67)	0.71 (0.09–5.42)	0.37 (0.03–4.26)	0.42 (0.03–5.00)

When examining food group intake by BMI category, the frequencies of intakes of red/processed meat and ready‐made meals increased significantly with increasing BMI category (Table 4). Furthermore, food group intakes varied by country of birth (Table 5). Whole‐grain foods were significantly more commonly consumed among women born in Norway and Western Europe/United States compared with Asian‐born women. Women from Norway and Asia had significantly lower intakes of fruits and berries than women born in Western Europe/United States and Eastern Europe. Fish was most commonly consumed among African‐born women, with a significantly more frequent intake than both Norwegian‐ and Asia‐born women. Beans and lentils were most commonly consumed by women born in Africa and South America. Red/processed meat was significantly more commonly consumed among Norwegian‐born women compared with women born in Eastern Europe. Norwegian women had the most frequent consumption of ready‐made meals and significantly more frequently than women born in Eastern Europe, Asia, and Africa. Sugar was significantly more often consumed by women born in Eastern Europe compared with women born in Norway.

**Table 4:** Mean weekly frequency of food group consumption by BMI category, *N* = 237

	BMI				
	<25	25–29.9	30–34.9	≥35	*p*‐value
	*N* = 102	*N* = 74	*N* = 37	*N* = 22	
Whole grain	6.7	6.3	7.2	6.3	0.46
Vegetables	9.0	8.2	9.7	7.8	0.10
Fruits and berries	7.8	7.5	8.0	7.2	0.79
Milk (low fat)	4.7	4.5	5.8	3.8	0.29
Fish	1.8	1.9	1.7	2.0	0.70
Beans and lentils	1.5	1.4	1.0	0.7	0.10
Vegetable oils	5.3	5.1	4.3	4.7	0.08
Red/processed meat	1.8	2.4	2.7	2.8	0.001
Ready‐made meals	0.8	1.1	1.0	1.5	0.03
Salt	3.6	3.5	3.7	4.0	0.22
Sugar	10.1	9.9	8.8	8.7	0.41
Saturated fat	2.8	3.5	3.8	3.1	0.12

**Table 5:** Mean weekly frequency of food group consumption by country of birth, *N* = 237

	Country of birth						p‐value
	Norway	Western Europe + USA	Eastern Europe	Asia	Africa	South America	
	*N* = 111	*N* = 14	*N* = 21	*N* = 57	*N* = 29	*N* = 5	
Whole grain	7.0	7.9	6.9	5.5	7.1	5.4	0.02
Vegetables	8.8	10.4	10.4	7.8	8.4	9.7	0.02
Fruits and berries	7.3	9.2	9.9	7.0	8.4	7.8	0.008
Milk (low fat)	4.7	3.8	5.5	4.0	6.3	5.0	0.17
Fish	1.6	1.9	1.9	1.7	2.8	1.7	<0.001
Beans and lentils	1.0	1.4	0.8	1.5	2.1	2.3	0.001
Vegetable oils	4.4	5.4	5.1	5.8	5.5	4.2	0.002
Red/processed meat	2.3	2.8	1.7	2.2	2.0	2.3	0.26
Ready‐made meals	1.3	1.2	0.7	0.8	0.6	0.5	0.003
Salt	3.5	3.9	3.1	3.4	4.6	3.5	0.53
Sugar	8.5	8.9	11.6	10.4	11.1	12.8	0.27
Saturated fat	3.8	3.0	3.4	2.4	2.8	2.1	0.02

## 4 DISCUSSION

This study assessed adherence to national dietary recommendations in a multi‐ethnic pregnant population prior to being diagnosed with GDM. A significantly higher proportion of non‐native Norwegian‐speaking women had a high HDS compared with native Norwegian‐speaking women. Participants with normal weight prepregnancy were more likely to have a high HDS. Even though the intake of food groups varied by country of birth, low adherence to some dietary recommendations can contribute to the development of GDM.

Poor adherence to national dietary recommendations was found in other studies among pregnant women (APrON & ENRICH Study Teams, 2017; Elvebakk, Mostad, Morkved, Salvesen, & Stafne, 2018; von Ruesten et al., 2014). According to the Norwegian guidelines for antenatal care (Directorate for Health and Social Affairs, 2005), women should receive information about the dietary recommendations at the beginning of pregnancy. Since the mean gestational week of our participants was 26, one can assume that they had received information about the dietary recommendations. Participants had low adherence to the recommendations for whole grains, vegetables, and fruits and berries, and a relatively low proportion adhered to the recommendations for intakes of fish, red/processed meat, and ready‐made meals. Interestingly, low adherence to similar specific recommendations was also found in the Norwegian Mother and Child Cohort Study among 47,011 pregnant women (von Ruesten et al., 2014). For instance, only 45% of pregnant women followed the recommendation for red and processed meats, and less than 10% followed the recommendations for vegetables and fatty fish (von Ruesten et al., 2014). Elvebakk et al. (2018) assessed dietary differences between women with and without GDM in a longitudinal study among 702 pregnant women. Similar to our findings, an excess intakes of red and processed meats and low intakes of vegetables and fatty fish were observed among the women (Elvebakk et al., 2018).

Maternal diet influences the risk of developing GDM. However, there is uncertainty about which dietary patterns constitute a higher risk of developing GDM (Schoenaker et al., 2016). A review found that higher dietary fat and lower carbohydrate intakes during pregnancy are associated with a higher risk for GDM, independent of pre‐pregnancy BMI (Morisset et al., 2010). In other studies, high consumption of processed meat, snacks, and fast foods and low consumption of vegetables before or during pregnancy were related to the development of GDM (Lamyian et al., 2017; Schoenaker et al., 2016), whereas diets high in rice, beans, and vegetables and low in full‐fat dairy products and sweets were inversely associated with GDM (Sartorelli, Zuccolotto, Crivellenti, & Franco, 2019). Shin et al. (2015) observed a connection between high consumption of refined grains, fat, and added sugars and low intake of fruits and vegetables during pregnancy with higher odds of developing GDM among 253 pregnant American women in the National Health and Nutrition Examination Survey (NAHNES). Taken together, the sample in the present study as a whole showed low adherence to some dietary recommendations that can contribute to the development of GDM.

Few studies have investigated ethnic differences in dietary quality of pregnant women (de Seymour et al., 2016; Sommer et al., 2013; Zhu et al., 2018). A cross‐sectional study among 757 pregnant women, of whom 59% were of non‐Western origin, found that non‐European ethnic origin and integration scores were associated with higher odds of having unhealthier dietary patterns (Sommer et al., 2013). In the present study, a significantly higher proportion of non‐native Norwegian‐speaking women had a high HDS compared with native Norwegian‐speaking women. This finding contradicts the findings of two systematic reviews of the literature on changes in dietary habits of selected ethnic groups in Europe, concluding that migration is associated with unhealthy dietary changes (Gilbert & Khokhar, 2008; Holmboe‐Ottesen & Wandel, 2012). South Asians living in Europe tend to adopt less healthy diets, characterized by increased intakes of energy, fat, and refined carbohydrates. A shift from vegetable to animal food sources is also commonly observed (Holmboe‐Ottesen & Wandel, 2012). In the present study, women born in Asia ate significantly lower quantities of whole grains than Norwegian‐born women. In addition, women born in Asia and Norway consumed significantly lower quantities of fruit and berries as well as fish compared with women born in Africa. Even though a high number of participants have been born in Norway to two foreign‐born parents, previous research indicates that young women appear to be strongly influenced by cultural traditions and family expectations with regard to food preparation and consumption (Ahlqvist & Wirfält, 2000; Mellin‐Olsen & Wandel, 2005; Thornton et al., 2006). The results of the present study confirm this, as cultural traditions might explain the healthier diet of women from an immigrant background compared with native Norwegian‐speaking women, whose diet is characterized by lower consumption of ready‐made meals and higher consumption of beans, lentils, and vegetables instead of red and processed meats.

Similar to previous studies, the results of this study show poorer adherence to national dietary recommendations among prepregnancy overweight and obese women compared with women of normal weight (APrON & ENRICH Study Teams, 2017; von Ruesten et al., 2014). For instance, obese women prepregnancy had lower scores for adherence to the Canadian Food Guide Recommendations than those who were of normal weight in a prospective cohort study among 1,630 pregnant Canadian women (APrON & ENRICH Study Teams, 2017). Obese women (BMI = 35–45) had a nine‐fold increase in the odds ratio of having a low HDS compared with normal weight women in an analysis that was adjusted for country of birth. These findings underline the need for more effective nutrition communication strategies targeting pregnant overweight and obese women to prevent the development of GDM.

Contrary to previous research (Doyle, Borrmann, Grosser, Razum, & Spallek, 2017; Sommer et al., 2013; Wesolowska et al., 2019; von Ruesten et al., 2014), poorer dietary scores were not associated with women's socio‐economic status and education in this study. It must be considered that the participants were more educated and had higher incomes than the average woman in Norway and might not be representative of the entire pregnant population in Norway. In the present study, 66.7% of women had pursued higher education (college or university), whereas the average in the Norwegian female population is 37% (Statistic Norway, 2019).

## 5 LIMITATIONS

The results of this study must be interpreted in the context of some limitations. The sample size of this study is limited, and thus, the results need to be interpreted with caution. The higher educational level of the sample compared with the average for Norwegian women as well as the small sample groups related to country of birth restrict the generalizability to the pregnant population in general. Social desirability might have biased the self‐report of dietary intake. In addition, participants were asked to report their diet in a FFQ prior to being diagnosed with GDM, and there might have been recall bias, as some women might have had to wait for approximately one week to be referred to the DOC and included in the study. In addition, some women might have received dietary advice after being diagnosed with GDM and prior to answering the FFQ which might have biased their response. When assessing dietary quality based on dietary scores derived from an FFQ, subjectivity is introduced regarding the selection and scoring of included components and cutoff points. No clear cutoff values for intakes of food groups can be established regarding the risk of chronic disease (Waijers, Feskens, & Ocke, 2007). Lastly, there was no assessment of whether participants had received advice regarding a healthy diet from health professionals during antenatal care.

## 6 CONCLUSIONS

This study evinced low adherence to national dietary recommendations among pregnant women. The adherence to national dietary recommendations was significantly lower among native Norwegian‐speaking women as compared to non‐native Norwegian‐speaking women. Although there were different food group intakes related to country of birth, participants had low adherence to dietary recommendations which may prevent the development of GDM. Given the increase in women with GDM and the emerging evidence that maternal diet is a modifiable risk factor for GDM, effective strategies for nutrition communication in antenatal care are urgently needed. According to the findings, these strategies should focus on promoting a healthy diet that is high in whole grains, fish, vegetables, and fruits and berries and low in red and processed meats and ready‐made meals.

## CONFLICT OF INTEREST

The authors do not have any conflicts of interest and have contributed to and approved the final version of this manuscript.

## ETHICAL APPROVAL

This study does not involve any human or animal testing. The Norwegian Social Science Data Services (id number: 2014/38942) and the patient privacy protection boards at the recruiting sites have reviewed and approved the study's protocol and procedures. The study conforms to the Declaration of Helsinki for human subjects.

## INFORMED CONSENT

Written informed consent was obtained from all participants.

## References

[fsn31248-bib-0001] Ahlqvist, M. , & Wirfält, E. (2000). Beliefs concerning dietary practices during pregnancy and lactation: A qualitative study among Iranian women residing in Sweden. Scandinavian Journal of Caring Sciences, 14(2), 105–111. Retrieved from http://search.ebscohost.com/login.aspx?direct=true&db=aph&AN=3970132&site=ehost-live 12035273

[fsn31248-bib-0002] APrON and ENRICH Study Teams . (2017). Adherence to Canada's food guide recommendations during pregnancy: Nutritional epidemiology and public health. Current Developments in Nutrition, 1(7), e000356 10.3945/cdn.116.000356 29955709 PMC5998351

[fsn31248-bib-0003] Bellamy, L. , Casas, J. P. , Hingorani, A. D. , & Williams, D. (2009). Type 2 diabetes mellitus after gestational diabetes: A systematic review and meta‐analysis. Lancet, 373(9677), 1773–1779. Retrieved from http://www.sciencedirect.com/science/article/pii/S0140673609607315 19465232 10.1016/S0140-6736(09)60731-5

[fsn31248-bib-0004] Borgen, I. , Garnweidner‐Holme, L. M. , Jacobsen, A. F. , Bjerkan, K. , Fayyad, S. , Joranger, P. , … Lukasse, M. (2017). Smartphone application for women with gestational diabetes mellitus: A study protocol for a multicentre randomised controlled trial. British Medical Journal Open, 7(3), e013117 10.1136/bmjopen-2016-013117 PMC5372027 28348183

[fsn31248-bib-0005] Bouthoorn, S. H. , Silva, L. M. , Murray, S. E. , Steegers, E. A. P. , Jaddoe, V. W. V. , Moll, H. , … Raat, H. (2015). Low‐educated women have an increased risk of gestational diabetes mellitus: The Generation R Study. Acta Diabetologica, 52(3), 445–452. 10.1007/s00592-014-0668-x 25344768

[fsn31248-bib-0006] Brosig, S. , Burggraf, C. , Teuber, R. , & Meier, T. (2018). Review of a priori dietary quality indices in relation to their construction criteria. Nutrition Reviews, 76(10), 747–764. 10.1093/nutrit/nuy027 30053192 PMC6130981

[fsn31248-bib-0007] de Seymour, J. , Chia, A. , Colega, M. , Jones, B. , McKenzie, E. , Shirong, C. , … Chong, M. (2016). Maternal dietary patterns and gestational diabetes mellitus in a multi‐ethnic Asian cohort: The GUSTO study. Nutrients, 8(9), 574 Retrieved from https://www.ncbi.nlm.nih.gov/pubmed/27657116. 10.3390/nu8090574 PMC5037559 27657116

[fsn31248-bib-0009] Directorate for Health and Social Affairs . (2005). A national clinical guideline for antenatal care. Short version. Retrieved from Oslo: http://www.helsedirektoratet.no/publikasjoner/national-clinical-guideline-for-antenatal-care-short-version/Publikasjoner/Inational-clinical-guideline-for-antenatal-care-short-version.pdf

[fsn31248-bib-0011] Doyle, I. M. , Borrmann, B. , Grosser, A. , Razum, O. , & Spallek, J. (2017). Determinants of dietary patterns and diet quality during pregnancy: A systematic review with narrative synthesis. Public Health Nutrition, 20(6), 1009–1028. 10.1017/s1368980016002937 27852338 PMC10261556

[fsn31248-bib-0012] Elvebakk, T. , Mostad, I. L. , Morkved, S. , Salvesen, K. A. , & Stafne, S. N. (2018). Dietary intakes and dietary quality during pregnancy in women with and without gestational diabetes mellitus—A Norwegian longitudinal study. Nutrients, 10(11). 10.3390/nu10111811 PMC6266178 30463394

[fsn31248-bib-0013] Garnweidner, L. M. , Sverre Pettersen, K. , & Mosdol, A. (2013). Experiences with nutrition‐related information during antenatal care of pregnant women of different ethnic backgrounds residing in the area of Oslo, Norway. Midwifery, 29(12), e130–e137. 10.1016/j.midw.2012.12.006 23481338

[fsn31248-bib-0014] Gilbert, P. , & Khokhar, S. (2008). Changing dietary habits of ethnic groups in Europe and implications for health. Nutrition Reviews, 66(4), 203–215. 10.1111/j.1753-4887.2008.00025.x 18366534

[fsn31248-bib-0015] Gluckman, P. D. , Hanson, M. A. , & Buklijas, T. (2010). A conceptual framework for the developmental origins of health and disease. Journal of Developmental Origins of Health and Disease, 1(1), 6–18. 10.1017/s2040174409990171 25142928

[fsn31248-bib-0016] Holmboe‐Ottesen, G. , & Wandel, M. (2012). Changes in dietary habits after migration and consequences for health: A focus on South Asians in Europe. Food & Nutrition Research, 56 10.3402/fnr.v56i0.18891 PMC3492807 23139649

[fsn31248-bib-0017] Jenum, A. K. , Diep, L. M. , Holmboe‐Ottesen, G. , Holme, I. M. , Kumar, B. N. , & Birkeland, K. I. (2012). Diabetes susceptibility in ethnic minority groups from Turkey, Vietnam, Sri Lanka and Pakistan compared with Norwegians—The association with adiposity is strongest for ethnic minority women. BMC Public Health, 12, 150 Retrieved from http://europepmc.org/abstract/MED/22380873 22380873 10.1186/1471-2458-12-150 PMC3315409

[fsn31248-bib-0018] Kim, C. (2014). Maternal outcomes and follow‐up after gestational diabetes mellitus. Diabetic Medicine: A Journal of the British Diabetic Association, 31(3), 292–301. Retrieved from https://www.ncbi.nlm.nih.gov/pubmed/24341443https://www.ncbi.nlm.nih.gov/pmc/PMC3944879/ 10.1111/dme.12382 24341443 PMC3944879

[fsn31248-bib-0020] Lamyian, M. , Hosseinpour‐Niazi, S. , Mirmiran, P. , Moghaddam Banaem, L. , Goshtasebi, A. , & Azizi, F. (2017). Pre‐pregnancy fast food consumption is associated with gestational diabetes mellitus among Tehranian women. Nutrients, 9(3). 10.3390/nu9030216 PMC5372879 28257029

[fsn31248-bib-0008] Legeforeningen, D. N. (2008). Veileder i Fødselshjelp. Diabetes i svangerskapet. Retrieved from https://legeforeningen.no/Fagmed/Norsk-gynekologisk-forening/Veileder-arkiv–utgatte-versjoner/veileder-i-fodselshjelp-2008/kapittel-8-diabetes-i-svangerskapet/

[fsn31248-bib-0021] Makgoba, M. , Savvidou, M. D. , & Steer, P. J. (2012). An analysis of the interrelationship between maternal age, body mass index and racial origin in the development of gestational diabetes mellitus. An International Journal of Obstetrics & Gynaecology, 119(3), 276–282. 10.1111/j.1471-0528.2011.03156.x 22044452

[fsn31248-bib-0022] Medical Birth Registry Norway . (2018). The medical birth registry Norway. Retrieved from http://statistikkbank.fhi.no/mfr/

[fsn31248-bib-0023] Mellin‐Olsen, T. , & Wandel, M. (2005). Changes in food habits among Pakistani immigrant women in Oslo, Norway. Ethnicity & Health, 10, 311–339. 10.1080/13557850500145238 16191730

[fsn31248-bib-0024] Morisset, A. S. , St‐Yves, A. , Veillette, J. , Weisnagel, S. J. , Tchernof, A. , & Robitaille, J. (2010). Prevention of gestational diabetes mellitus: A review of studies on weight management. Diabetes/Metabolism Research and Reviews, 26(1), 17–25. 10.1002/dmrr.1053 19943327

[fsn31248-bib-0025] Neiger, R. (2017). Long‐term effects of pregnancy complications on maternal health: A review. Journal of Clinical Medicine, 6(8), 76 Retrieved from , https://www.ncbi.nlm.nih.gov/pubmed/28749442https://www.ncbi.nlm.nih.gov/pmc/PMC5575578/. 10.3390/jcm6080076 PMC5575578 28749442

[fsn31248-bib-0027] Olmedo‐Requena, R. , Gomez‐Fernandez, J. , Amezcua‐Prieto, C. , Mozas‐Moreno, J. , Khan, K. S. , & Jimenez‐Moleon, J. J. (2019). Pre‐pregnancy adherence to the mediterranean diet and gestational diabetes mellitus: A case‐control study. Nutrients, 11(5). 10.3390/nu11051003 PMC6566892 31052474

[fsn31248-bib-0045] Øverby, N. C. , Hillesund, E. R. , Sagedal, L. R. , Vistad, I. , & Bere, E. (2015). The Fit for Delivery study: Rationale for the recommendations and test‐retest reliability of a dietary score measuring adherence to 10 specific recommendations for prevention of excessive weight gain during pregnancy. Maternal & Child Nutrition, 11(1), 20–32. Retrieved from https://onlinelibrary.wiley.com/doi/abs/10.1111/mcn.12026. 10.1111/mcn.12026 23241065 PMC6860359

[fsn31248-bib-0028] Rani, P. R. , & Begum, J. (2016). Screening and diagnosis of gestational diabetes mellitus, where do we stand. Journal of Clinical and Diagnostic Research, 10(4), Qe01–Qe04. 10.7860/jcdr/2016/17588.7689 PMC4866200 27190902

[fsn31248-bib-0029] Sartorelli, D. S. , Zuccolotto, D. C. C. , Crivellenti, L. C. , & Franco, L. J. (2019). Dietary patterns during pregnancy derived by reduced‐rank regression and their association with gestational diabetes mellitus. Nutrition, 60, 191–196. 10.1016/j.nut.2018.10.008 30612039

[fsn31248-bib-0030] Schneider, S. , Hoeft, B. , Freerksen, N. , Fischer, B. , Roehrig, S. , Yamamoto, S. , & Maul, H. (2011). Neonatal complications and risk factors among women with gestational diabetes mellitus. Acta Obstetricia et Gynecologica Scandinavica, 90(3), 231–237. 10.1111/j.1600-0412.2010.01040.x 21306307

[fsn31248-bib-0031] Schoenaker, D. A. , Mishra, G. D. , Callaway, L. K. , & Soedamah‐Muthu, S. S. (2016). The role of energy, nutrients, foods, and dietary patterns in the development of gestational diabetes mellitus: A systematic review of observational studies. Diabetes Care, 39(1), 16–23. 10.2337/dc15-0540 26696657

[fsn31248-bib-0032] Shah, B. R. , Retnakaran, R. , & Booth, G. L. (2008). Increased risk of cardiovascular disease in young women following gestational diabetes mellitus. Diabetes Care, 31(8), 1668–1669. Retrieved from , https://www.ncbi.nlm.nih.gov/pubmed/18487472https://www.ncbi.nlm.nih.gov/pmc/PMC2494649/. 10.2337/dc08-0706 18487472 PMC2494649

[fsn31248-bib-0033] Shapiro, A. L. B. , Kaar, J. L. , Crume, T. L. , Starling, A. P. , Siega‐Riz, A. M. , Ringham, B. M. , … Dabelea, D. (2016). Maternal diet quality in pregnancy and neonatal adiposity: The Healthy Start Study. International Journal of Obesity (London), 40(7), 1056–1062. 10.1038/ijo.2016.79 PMC5356926 27133623

[fsn31248-bib-0034] Shepherd, E. , Gomersall, J. C. , Tieu, J. , Han, S. , Crowther, C. A. , & Middleton, P. (2017). Combined diet and exercise interventions for preventing gestational diabetes mellitus. Cochrane Database Systematic Review, 11, Cd010443 10.1002/14651858.CD010443.pub3 PMC6485974 29129039

[fsn31248-bib-0035] Shin, D. , Lee, K. W. , & Song, W. O. (2015). Dietary patterns during pregnancy are associated with risk of gestational diabetes mellitus. Nutrients, 7(11), 9369–9382. 10.3390/nu7115472 26569302 PMC4663600

[fsn31248-bib-0036] Sommer, C. , Sletner, L. , Jenum, A. K. , Morkrid, K. , Andersen, L. F. , Birkeland, K. I. , & Mosdol, A. (2013). Ethnic differences in maternal dietary patterns are largely explained by socio‐economic score and integration score: A population‐based study. Food & Nutrition Research, 57 10.3402/fnr.v57i0.21164 PMC3707086 23843779

[fsn31248-bib-0037] Statistic Norway . (2019). Educational attainment of the population. Retrieved from https://www.ssb.no/en/utdanning/statistikker/utniv

[fsn31248-bib-0038] Szwajcer, E. M. , Hiddink, G. J. , Koelen, M. A. , & van Woerkum, C. M. (2005). Nutrition‐related information‐seeking behaviours before and throughout the course of pregnancy: Consequences for nutrition communication. European Journal of Clinical Nutrition, 59, 57–65. 10.1038/sj.ejcn.1602175 16052197

[fsn31248-bib-0039] Thornton, P. L. , Kieffer, E. C. , Salabarria‐Pena, Y. , Odoms‐Young, A. , Willis, S. K. , Kim, H. , & Salinas, M. A. (2006). Weight, diet, and physical activity‐related beliefs and practices among pregnant and postpartum Latino women: The role of social support. Maternal and Child Health Journal, 10(1), 95–104. Retrieved from http://www.ncbi.nlm.nih.gov/pubmed/16534660. 10.1007/s10995-005-0025-3 16534660

[fsn31248-bib-0040] von Ruesten, A. , Brantsaeter, A. L. , Haugen, M. , Meltzer, H. M. , Mehlig, K. , Winkvist, A. , & Lissner, L. (2014). Adherence of pregnant women to Nordic dietary guidelines in relation to postpartum weight retention: Results from the Norwegian Mother and Child Cohort Study. BMC Public Health, 14, 75 10.1186/1471-2458-14-75 24456804 PMC3908932

[fsn31248-bib-0041] Waijers, P. M. , Feskens, E. J. , & Ocke, M. C. (2007). A critical review of predefined diet quality scores. British Journal of Nutrition, 97(2), 219–231. 10.1017/s0007114507250421 17298689

[fsn31248-bib-0042] Wesołowska, E. , Jankowska, A. , Trafalska, E. , Kałużny, P. , Grzesiak, M. , Dominowska, J. , … Polańska, K. (2019). Sociodemographic, lifestyle, environmental and pregnancy‐related determinants of dietary patterns during pregnancy. International Journal of Environmental Research and Public Health, 16(5). 10.3390/ijerph16050754 PMC6427254 30832307

[fsn31248-bib-0043] World Health Organization . (2013). Diagnostic criteria and classification of hyperglycaemia first detected in pregnancy. Retrieved from Geneva, Switzerland: https://apps.who.int/iris/bitstream/handle/10665/85975/WHO_NMH_MND_13.2_eng.pdf;jsessionxml:id=15530B32F54634921C2879F9ADEB71B4?sequence=1 24199271

[fsn31248-bib-0044] Zhu, Y. , Hedderson, M. M. , Sridhar, S. , Xu, F. , Feng, J. , & Ferrara, A. (2018). Poor diet quality in pregnancy is associated with increased risk of excess fetal growth: A prospective multi‐racial/ethnic cohort study. International Journal of Epidemiology, 48, 423–432. 10.1093/ije/dyy285 PMC6469312 30590563

